# Preparation and Characterization of Chitin Benzoic Acid Esters 

**DOI:** 10.3390/molecules16043029

**Published:** 2011-04-08

**Authors:** Lok Ranjan Bhatt, Bo Mi Kim, Kim Hyun, Geu Beum Kwak, Chai Ho Lee, Kyu Yun Chai

**Affiliations:** Department of Bionanochemistry, School of Natural Sciences, Wonkwang University, Iksan, Chonbuk 570-749, Korea

**Keywords:** acylation, chitin benzoate, benzoic acid, trifluoroacetic anhydride, phosphoric acid

## Abstract

Chitin benzoic acid esters were prepared using a phosphoryl mixed anhydride method. The products were characterized by ^1^H-NMR and FT-IR spectroscopy. FT-IR analysis revealed that the degree of *O*-acyl substitution of the products was in a range of 1.17–1.83. Morphological surface changes in the parent molecule due to the introduction of benzoic acid moieties were observed by scanning electron microscopy. The surface of the products was porous, in contrast to the sheet-shape of the parent molecules. The solubility of the products, which improved with increased degree of acid substitution, was tested in various organic solvents.

## 1. Introduction

**Scheme 1:**
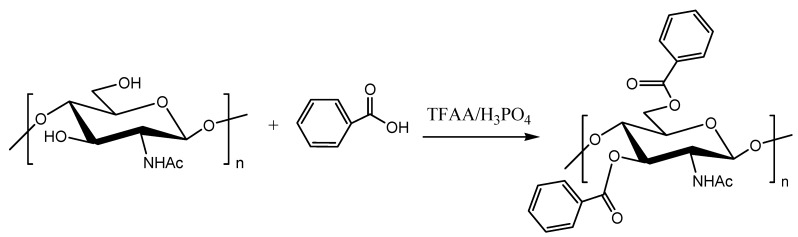
TFAA/H_3_PO_4_ promoted acylation of chitin with benzoic acid.

Chitin is a naturally abundant biocompatible, biodegradable, and bioactive polymer [1,2]. These characteristics have attracted great interest for its broad spectrum of applications in diverse areas such as biomedicine, cosmetics, pharmaceuticals, food processing, and others [1,2,3,4]. The structure of chitin is similar to that of cellulose, the only difference being the replacement of the hydroxyl group at the C-2 position of cellulose by an acetamido group in chitin [1]. Partially deacetylated chitin is known as chitosan, which contains copolymers of both glucosamine and *N*-acetylglucosamine. Chitin and chitosan are interesting polysaccharides because of the presence of the (acetyl)-amino and hydroxyl functional groups, which could be suitably modified to obtain new materials with desired properties and functions [1,4]. However, the insolubility of chitin in common organic solvents remains an obstacle for its commercial utilization. The solubility and processability is improved when the native chitin is modified by various chemical modifications, such as acylation [1,5,6]. Several studies have been carried on esterification of chitin molecules using acid anhydrides [7], acyl chlorides [8,9] and *p*-toluenesulfonyl chloride and carboxylic acid [10]. The use of trifluoroacetic anhydride as a promoter for esterification of a variety of *O*-acylated chitin derivatives is also noted [5,6]. However, little information is available on the acylation of chitin using aromatic carboxylic acids. Previously chitin 6-*O*-ethyl benzoate was prepared by the reaction of tosyl chloride with chitin in a DMAc/LiCl solvent system, followed by the reaction with the sodium salt of ethyl *p*-hydroxybenzoate [11]. Similarly, variously benzoylated chitins were prepared by reacting chitin with benzoyl chloride and methanesulfonic acid [8]. The trifluoroacetic anhydride/phosphoric acid (TFAA/H_3_PO_4_) reaction system is an efficient coupling agent for the direct *O*-acylation of phenolic hydroxy groups with free carboxylic acids [12]. This method has been employed for the preparation of a range of aryl carboxylate esters [12], and more recently for the preparation of chitin butyrate [13]. In this work, we report the TFAA/H_3_PO_4_ promoted synthesis of chitin benzoates by reacting chitin with benzoic acid (Scheme 1, Table 1). 

**Table 1:** Preparation of chitin benzoates from chitin and benzoic acid in the presence of TFAA/H_3_PO_4_.

Product	Chitin (g)	Benzoic acid (g)	TFAA (mL)	H_3_PO_4_ (mL)	Yield (g)	DS^a^
**1**	1.0	0.60	2.80	0.34	0.80	1.17
**2**	1.0	2.40	5.60	0.34	1.00	1.83
**3**	1.0	4.80	2.80	0.34	1.40	1.60

^a^ Degree of substitution based on FT-IR analysis.

## 2. Results and Discussion

The acylation of chitin was carried out by reacting chitin with benzoic acid in the presence of TFAA/H_3_PO_4_. When the chitin flakes were used as received from the manufacturer, the reaction mixture was viscous, even after extended period, especially for **1**; a dark reaction solution was obtained within 5 h when powdered chitin was employed. Nevertheless, some unreacted chitin still remained in the reaction mixtures, in particular for **1**. Moreover, miscibility of the solution of benzoic acid and TFAA with phosphoric acid is an important factor for the success of the reaction when a lower mole ratio of benzoic acid and TFAA to chitin is used.

The degree of the acid substitution (DS) of the products was estimated by FT-IR spectroscopy by comparing the absorption ratios of two carbonyl absorptions; amide I (~1,662 cm^−1^) and ester (~1,725 cm^−1^), as described elsewhere [14]. For this, the peak height of above two absorption bands was measured by using a straight baseline drawn from ~1,845 to ~900 cm^−1^. The DS of the obtained products was between 1.17–1.83, depending on the mole ratio of benzoic acid and TFAA added to chitin (Table 1). 

**Figure 1:**
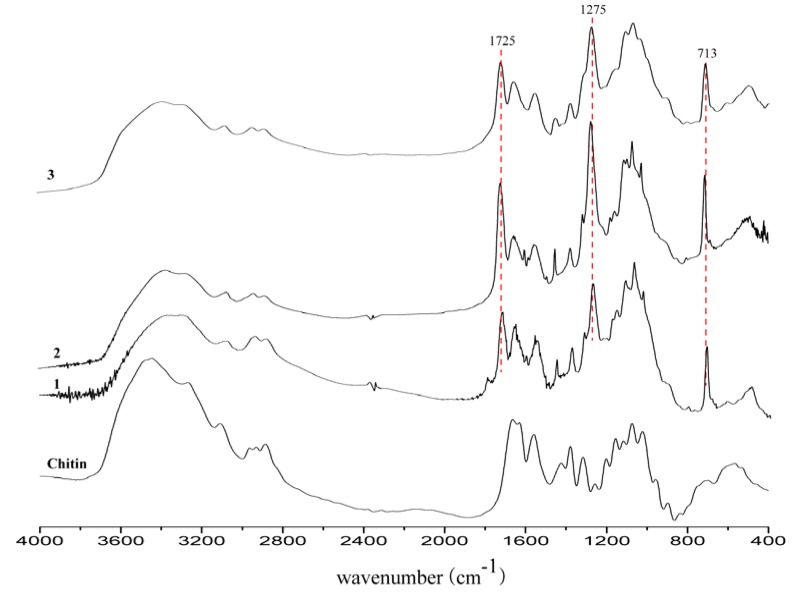
FT-IR spectra of chitin benzoic acid esters.

The results showed that an appropriate molar ratio of both TFAA and benzoic acid is required in order to get the optimum conditions. The products, recovered as slurry by diethyl ether precipitation, were soluble in a mixture of ethanol/acetone, but the air-dried products, except **2** (slightly soluble in ethanol and methanol), were insoluble in the above solvents. The solubility of the products improved, as the substitution of added benzoic acid increased (Table 2). Product **2 **had the highest DS and was readily soluble in dimethylsulfoxide (DMSO), *N,N*-dimethylformamide (DMF), benzyl alcohol (BnOH), formic acid (FA) and slightly soluble in methanol (MeOH), ethanol (EtOH) and acetic acid. Previously, chitin benzoate soluble in multiple organic solvents was prepared by Somorin and coworkers [8] by reacting excess benzoyl chloride with chitin (10:1) in methanesulfonic acid solution. However, the present system gave rise the chitin benzoates under mild and simple reaction conditions and using a lower molar ratio of benzoic acid and TFAA to chitin. 

**Table 2:** Solubility of chitin benzoic acid esters in selected organic solvents.^ a^

Product	Solubility^ a^							
	DMSO	DMF	BnOH	MeOH	EtOH	Acetone	FA	AcOH
**1**	S	SS	S	IS	IS	IS	S	IS
**2**	S	S	S	SS	SS	IS	S	SS
**3**	S	S	S	IS	IS	IS	S	IS

^a^ S = soluble; SS = Slightly soluble; IS = Insoluble

The FT-IR spectra (Figure 1) of the products showed the presence of amide absorption bands around 1662 cm^−1^ and 1557 cm^−1^, as for the parent molecule. The appearance of new absorption bands around 1725 cm^−1^ (C=O) and 1275 cm^−1 ^(C–O), low intensity absorption bands around 3,000 cm^−1^ (aromatic ring C–H stretching), and intense absorption bands around 713 cm^−1^ (out of plane C–H bending) for the products indicated the inclusion of benzoic acid moieties into the parent molecules. The strong absorption bands around 1,725 cm^−1^ and 1,275 cm^−1^ (benzoic acid ester) and the decrease in the absorption band around 3,400-3,500 cm^−1^ (OH group) in the FT-IR spectra of the products confirmed the *O*-acylation of chitin [8,9]. However, shifting of the amide I band (1,662 cm^−1^) towards lower frequency (1,655-1,659 cm^−1^), which can be attributed to the overlap of acetamide/amino groups with benzoic acid, indicated the partial *N*-acylation of parent molecules [15]. Moreover, appearance of a very weak absorption band (shoulder) around 1,710 cm^−1^, in particular for **1**, also supported the above fact. Similar *N*-acylation of the acetamide and/or *N, N*-diacyl substitutions were previously reported for the acylation of chitosan [16] and chitin [5,9]. However, the rate and extent of *O*-acylation seems to be much greater than *N*-acylation.

**Figure 2:**
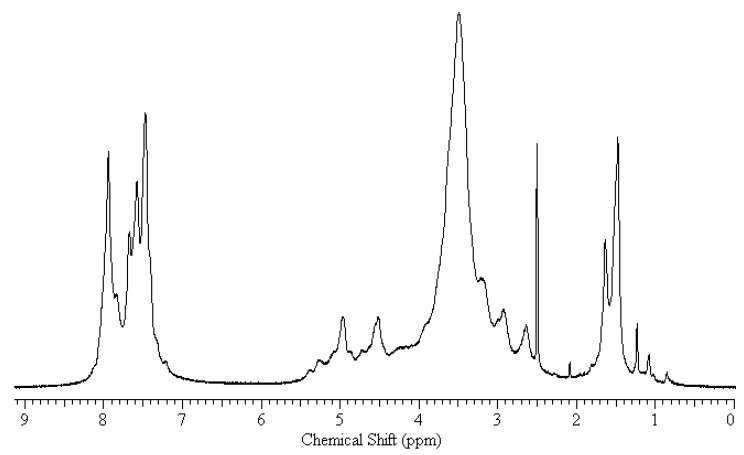
^1^H-NMR spectra of chitin benzoic acid ester (**3).**

The products were also characterized by ^1^H-NMR spectroscopy (Figure 2). The spectra, though their reolution was poor showed characteristic proton signals of chitin backbone (*δ* 3.14–4.97), N–H proton (*δ* 7.47–7.73 overlapped with phenyl protons) and acetamido methyl protons (~*δ* 1.6–1.8). Additional signals for phenyl protons were detected around *δ* 7.47–7.96. These signals indicated the inclusion of benzoic acid into the resulting product. Signals appeared around *δ* 1.0–1.5 seems impurities.

**Figure 3:**
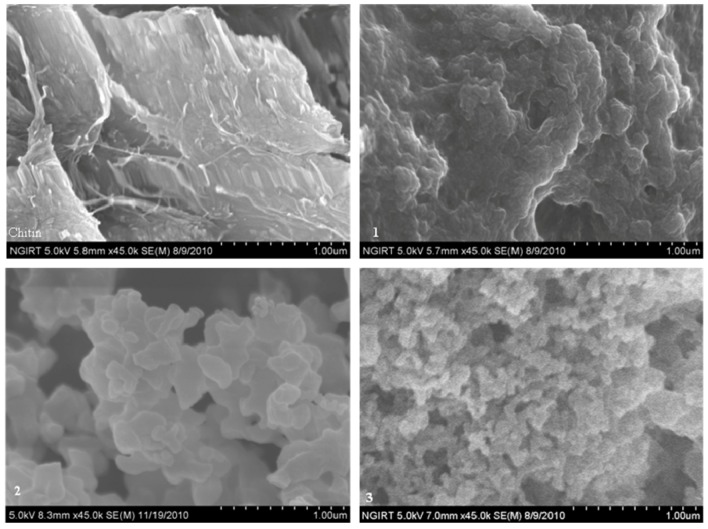
SEM images of chitin and chitin benzoic acid esters.

The surface morphology of chitin and the products was observed using SEM. The morphological surface changs in the parent molecule due to the introduction of the benzoic acid ester moieties were clearly visible (Figure 3). The surface of the products was porous, in comparison to the sheet-like appearance of the parent molecules. In particular, compound **2** and **3** exhibited highly porous surface areas with some finger like projections. Despite the porous surface area, the lower solubility of **3** (as compared to **2**) is likely due to its lower DS value. Additionally, the higher surface porosity of **3** observed in SEM could probably be due to the polydisperse nature of the size of the molecules (presence of morphologically differentiated domains with different molecular sizes).

## 3. Experimental

### 3.1. General

^1^H-NMR spectra of the products were recorded at room temperature (20 ºC) on a JEOL JNM-ECP 500 NMR spectrometer (500 MHz) using 5 mm diameter tubes. Samples were dissolved in DMSO-*d*_6_ at the concentration of 15 mg/mL. FT-IR spectra were recorded on a Shimadzu Prestige-21 FT-IR spectrometer, as described previously [14]. The surface morphology of chitin and the products was observed by scanning electron microscopy, using a S–4800 instrument (Hitachi, Japan). Samples were analyzed at an accelerating voltage of 5 kV and 5,000–45,000× magnification. The solubility of the sample was determined by taking 10 mg of sample into 2 mL of tested solvent in a closed vial for 24 h at room temperature with continuous shaking. The solubility was determined by visual observations.

### 3.2. Materials

Chitin was purchased from YB Bio (Gyeongbuk, Korea), trifluoroacetic anhydride from Acros Organics (New Jersey, USA); Dimethyl sulfoxide-*d*_6_ (DMSO-*d*_6_) from Cambridge Isotope Laboratories, Inc, (Andover, MA, USA); 85% phosphoric acid from Sigma Aldrich (St, Louis, MO, USA); and benzoic acid from Samchaun Pure Chemical Co. Ltd., (Pyeongtaek, Gyeonggi, Korea). All other reagents were of analytical grade. Chitin flakes were dried for 1 h at 50°C and ground into powder before use.

### 3.3. Synthesis

Chitin benzoates were prepared as described previously [13,17]. In brief, trifluoroacetic anhydride (TFAA) and benzoic acid were initially combined and stirred at 40 ºC until complete dissolution. The resulting solution was then cooled (ice bath) and mixed with 85% phosphoric acid before chitin was added (Table 1). The reaction mixture was then stirred at 50 °C for up to 5 h. The reaction solution when cooled to room temperature was mixed with cold ethanol (30 mL) before filtration. The filtrate was concentrated under reduced pressure (rotary evaporator, < 30 °C) to a syrup, which was dissolved in a mixture of ethanol/acetone and the product was obtained by precipitation with diethyl ether and recovered by filtration. This process was repeated until no color appeared on washing. Air-dried products were further dried in a vacuum oven before analysis.

## 4. Conclusions

The presence of rigid crystalline domains formed by intra and/or inter-molecular hydrogen bonding is considered to be responsible for the poor solubility of chitin [18] which is a major obstacle for its industrial application. TFAA/H_3_PO_4_ mediated *O*-acylation of chitin with benzoic acid provided chitin benzoates with improved solubility in multiple organic solvents. The products could find application as coating and packing materials in plastic and separation technology.
